# Functional Polymer Nanocarriers for Photodynamic Therapy

**DOI:** 10.3390/ph11040133

**Published:** 2018-11-30

**Authors:** Tuanwei Li, Lifeng Yan

**Affiliations:** CAS Key Laboratory of Soft Matter Chemistry, iChEM, and Department of Chemical Physics, University of Science and Technology of China, Hefei 230026, China; lituanwe@mail.ustc.edu.cn

**Keywords:** photodynamic therapy, nanocarriers, photosensitizers, hypoxia

## Abstract

Photodynamic therapy (PDT) is an appealing therapeutic modality in management of some solid tumors and other diseases for its minimal invasion and non-systemic toxicity. However, the hydrophobicity and non-selectivity of the photosensitizers, inherent serious hypoxia of tumor tissues and limited penetration depth of light restrict PDT further applications in clinic. Functional polymer nanoparticles can be used as a nanocarrier for accurate PDT. Here, we elucidate the mechanism and application of PDT in cancer treatments, and then review some strategies to administer the biodistribution and activation of photosensitizers (PSs) to ameliorate or utilize the tumor hypoxic microenvironment to enhance the photodynamic therapy effect.

## 1. Introduction

**Scheme 1:**
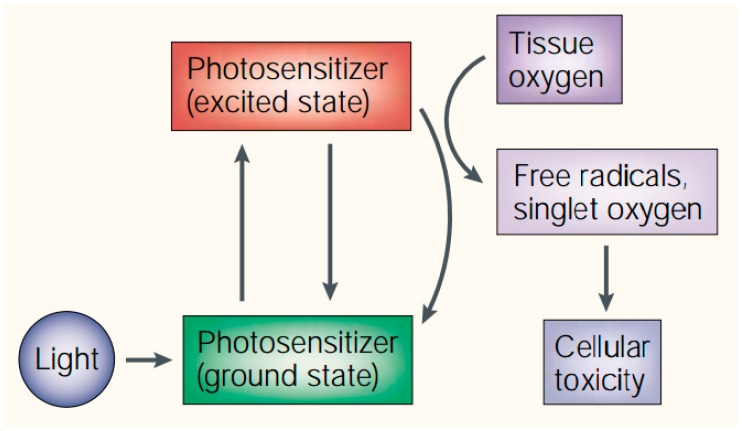
Mechanism of action of photodynamic therapy (PDT). Reproduced with permission from [8].

Photodynamic therapy (PDT) is a non-systemic therapeutic procedure and can function only when combining with three nontoxic individual components (the light-activated photosensitizer, specific light source and molecular oxygen) [1,2,3,4,5,6,7,8]. The photosensitizers (PS), once activated by light of matched wavelengths and dose, can undergo chemical reaction through two types of mechanisms [9,10]. In type I reaction, free radicals and radical ions are formed through the electron/hydrogen transfer process between photosensitizers and substrate molecules [11]. In type II mechanism (the dominant process in PDT), highly cytotoxic singlet oxygen species (^1^O_2_) are produced through the energy transfer process from PS to molecular oxygen (Scheme 1) [9,12]. The optimal effective action radius of the ^1^O_2_ is in 20 nanometers due to its high reactivity, which means the PDT is a highly localized treatment [5,13,14,15,16,17,18,19].

The PDT is of dual selectivity for the preferential accumulation of PSs in neoplastic lesions and precise spatiotemporal control of the light. Therefore, PDT has outstanding advantages in rapidly destroying the primary tumor and avoiding unnecessary side effects to healthy tissues [20]. As a noninvasive therapeutic mode, PDT is a more secure, convenient, and less painful therapeutic option and can significantly improve the life quality of patients [21]. Currently, photodynamic therapy has become a new intriguing treatment modality in the field of age-related macular degeneration (AMD) [22], polypoidal choroidal vasculopathy (PCV) [23,24,25,26,27,28,29,30], non-melanoma skin cancer, oral premalignant lesions, head and neck squamous cell carcinoma dermatology, and dentistry [23,31,32,33,34,35]. Additionally, the PDT can also interrupt the vessel integrity and promote the delivery efficiency of drug load [36,37,38].

However, conventional photodynamic therapy also suffers from several dilemmas, including the light penetration depth in tissues and activation efficiency to PSs [39,40,41], oxygen reliance, and oxygen consumption during PDT [42,43,44], biodistribution of PSs in the targeted site and persistent skin and eyes photosensitivity [9,17,45,46,47,48,49,50,51]. Therefore, great efforts have been devoted to manage the distribution of PSs, increase penetration depth for deep tissue treatment, and improve oxygen supply of the tumor tissue [1,40,41,43,52,53,54,55,56,57,58,59].

Nanocarriers, particularly functional polymer nanocarriers, offer unique therapeutic application platforms for PDT because of their controllable size and shape, and extensible functionalities [3,6,47,60,61]. Numerous reviews have been published to discuss the conventional design concepts for efficient delivery and specific activation of PSs, introduce the revolutionary strategies for deep tumor treatment and summarize the comprehensive application of nanoparticles for enhanced PDT [6,47,61,62,63]. On this basis, this review is devoted to functional polymer nanocarrier platforms which can enhance PDT due to their specific tumor targeting or stimulus responsiveness. We will attempt to provide an overview of the nanocarriers by focusing on the work on administrating the biodistribution and activation of PSs, improving the tumor hypoxic microenvironment, as well as extending to the combination therapy of photodynamic therapy and other treatments.

## 2. Administration of PSs

The biodistribution and photochemical activity of PSs are important parameters during PDT [48,60]. In this part of the review, we will introduce how to administer the photosensitizers with functional polymer nanocarriers.

### 2.1. Administrate the Biodistribution of PSs via Targeting

**Figure 1:**
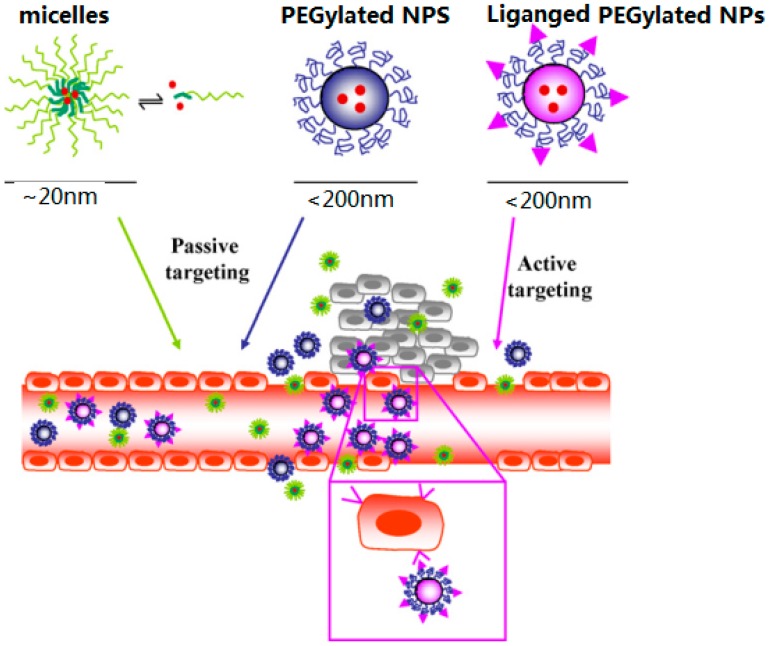
Schematic presentation of passive and active PDT. Reprinted from International Journal of Pharmaceutics, 392, Fabienne Danhier et al., Active and passive tumor targeting of a novel poorly-soluble cyclin-dependent kinase inhibitor, JNJ-7706621, 20–28., Copyright (2010), with permission from Elsevier [67].

The biodistribution of the photosensitizers in vivo is a clinically intractable issue for PDT [19]. After entering the bloodstream, drugs can relocate in the body as a function of time and affect the PDT [64,65]. It can, thus, be generally expected that the PSs can selectively concentrate in the targeted tumor site effectively and minimally reside in non-targeted normal tissue. Functional polymer nanocarriers can enhance the solubility of hydrophobic PSs and prevent their aggregation in blood. Meanwhile, the drug delivery system can administrate the accumulation of hydrophobic drug by specific target recognition and/or enhanced permeability and retention effect (EPR) of solid tumors. Hence, the nanocarriers have been promising platforms to modulate the biodistribution of PSs (Figure 1) [66].

#### 2.1.1. Passive Targeting

The passive target is a common strategy to increase the specific accumulation in target tissues [15,63]. By optimizing the physicochemical properties, the polymer nanocarriers can accumulate in the tumor tissues selectively through the EPR effect due to their prolonged circulation time in the blood [3]. Many nanocarrier systems based on biodegradable polymer have been used in PDT. By incorporating hydrophobic PSs into nanoparticles, and the non-aggregated PSs revealed higher ^1^O_2_ quantum yield than their aggregates [68,69]. The nanoparticles can selectively accumulate within the target tumor tissue and enhance the light-dark toxicity ratio [70,71].

**Figure 2:**
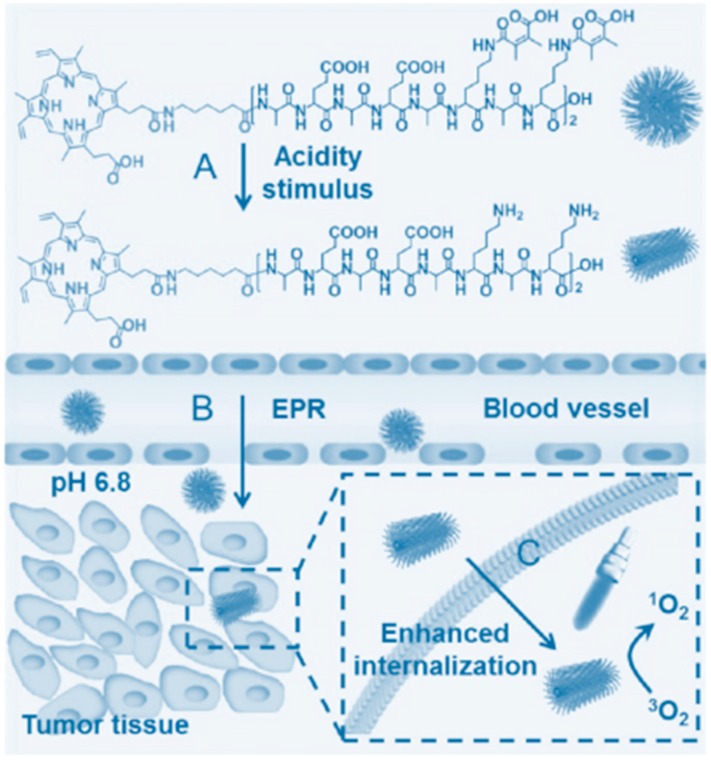
Schematic illustration of acidity-triggered geometrical shape switch and enhanced PDT. Copyright (2017) American Chemical Society [76].

The geometrical shape of nanocarrier has a powerful impact on cellular internalization [72,73]. Rod-like nanoparticles can facilitate the cellular uptake than spherical nanoparticles in general [74,75]. Tumor-triggered geometrical-shape-switched nanoparticles based on chimeric peptide (PEAK-DMA) was reported by Han et al. [76]. The chimeric peptide can self-assemble into spherical nanoparticles in neutral solution while forming short rod-like nanoparticles when triggered by tumor extracellular acidity. This nanocarriers with geometrical shape switch can enhance the targeted PDT efficacy and reduce cytotoxicity to normal tissue (Figure 2).

The surface property of the nanoparticles is another important factor for passive target. Nanoparticles with negatively-charged surfaces are effective in evading the reticuloendothelial system (RES) and prolong blood circulation [51,77,78,79]. However, the electro-positivity is much better to accelerate cellular internalization for the electro-negativity of cell membranes. Liu et al. developed a charge reversible upconversion nanocarrier loading with Chlorin e6 (Ce6) to achieve tissue-penetrating PDT [59]. The nanocarrier can expose positively charged naked surface by removing the dimethylmaleic acid (DMMA) groups under the slightly acidic tumor microenvironment and significantly enhance cell uptake.

#### 2.1.2. Active Targeting

Active targeting, distinguishing from passive targeting, delivers PSs to cancer tissue specificity based on molecular recognition [19]. During positive targeting, specific ligands of the carriers can recognize and then bind to appropriate receptors overexpressed only at the target site. In this way, active targeting can guarantee the specific accumulation of nanoparticles in tumor tissues and enhance the specificity of PDT. The targeting moieties, such as peptides [66,80,81], aptamers [82], and proteins have been applied to target tumor vasculature [83,84], tumor cells [85], and subcellular organelles [45,46].

The vasculature targeting performs its function in two ways: destroying the vasculature directly or enhancing the delivery capacity of PSs to the tumor. Destruction of the endothelium, which shows no differences among different types of solid tumors, can suppress cancer growth and metastatic ability by cutting off the supply of oxygen and nutrients [15]. Additionally, vasculature targeting can significantly increase nanocarrier accumulation in tumor tissue by interrupting the vessel integrity.

Vascular endothelial growth factor (VEGF) and its receptor are important hallmarks overexpressed on the tumor cells. Both of them can be designed as main antiangiogenic targets [56,86]. Combining with PDT, VEGFR has been a validated molecular target for head and neck squamous cell carcinoma [34,86,87,88,89,90].

Vascular cell adhesion molecule-1 (VCAM-1) is bound up with tumor cell adhesion and metastasis [84,89]. Fu et al. designed a targeted nanodrug (PVQ) by loading the PSs and VCAM-1 binding peptides with conjugated water-dispersible colloidal [84], indicated that the PVQ can target VCAM-1 expressing tumor cells selectively while have no obvious preferences to the normal cells [84].

**Figure 3:**
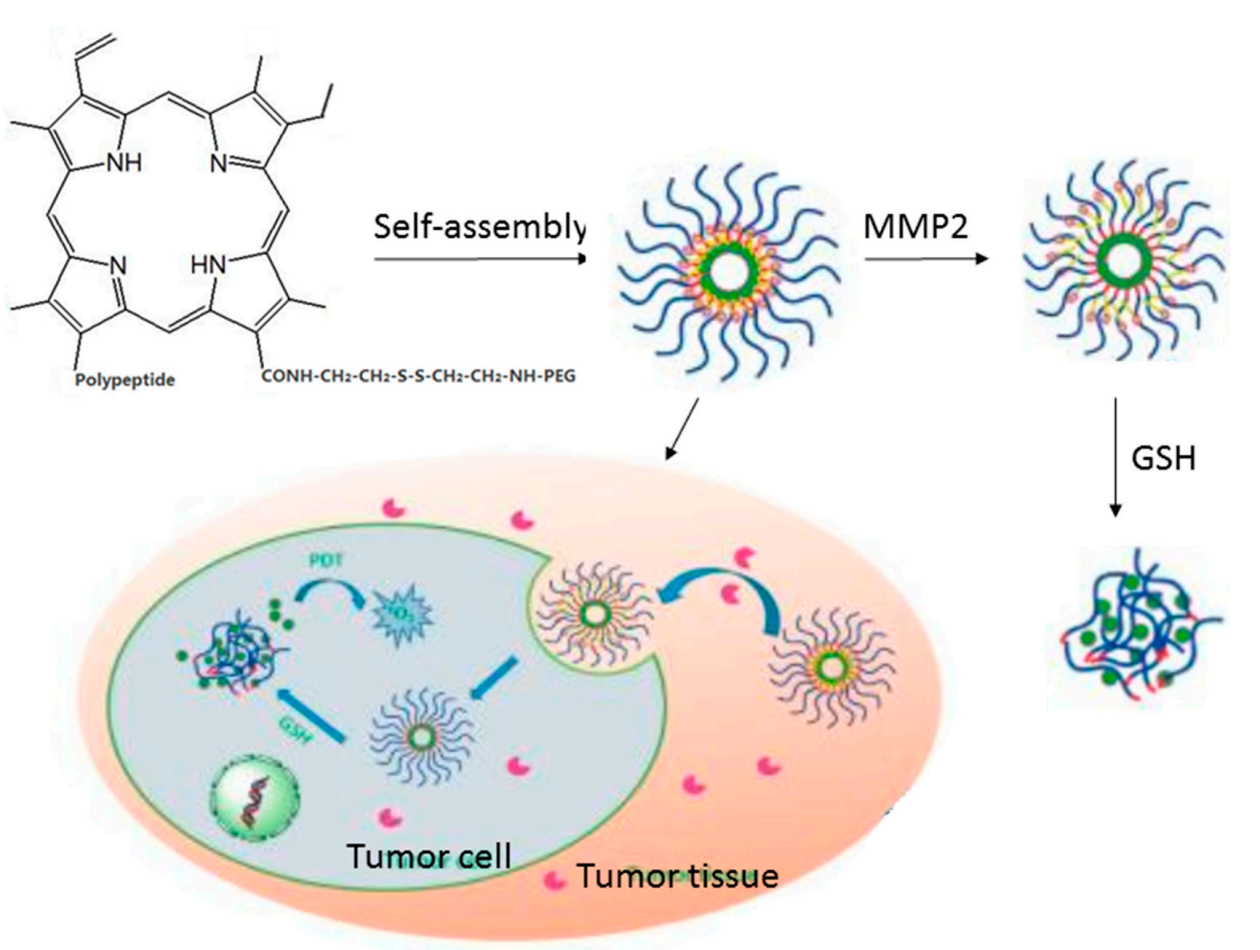
Schematic illustration of the preparation of the PEG-SS-Ce6-MMP2 NPs and Their applications in vivo. Copyright (2016) American Chemical Society [52].

The matrix metalloproteinases (MMPs) are the drivers of angiogenesis and metastasis, and overexpressed on endothelial tumor cells [89,91]. Cui et al. developed a redox-responsive nanocarriers for MMP2-targeting photodynamic therapeutic (Figure 3) [52]. The model PS was modified with hydrophobic polypeptide and hydrophilic polyethylene glycol (PEG) via MMP2-cleavable conjugate structure and GSH-responsive disulfide linker, respectively. After forming nanoparticles via self-assembly in aqueous solution, the nanocarrier can specifically accumulate in the areas of the tumor owing to the EPR effect and MMP2 targeting. After that, the free Ce6 was delivery in tumor cells triggered by GSH and significantly improved PDT efficiency [52].

The RGD sequence can be recognized by dozens of integrins and endow prodrug with targeting function when be integrated into nanocarriers [92,93,94,95,96]. α_v_β_3_ integrin is closely related to the endothelial cell migration by impacting calcium-dependent signaling pathway and it is overexpressed mainly on neovascular endothelial cells [19,89]. Xie et al. designed a tumor vasculature targeted PDT nanocarriers using RGD-modified ferritin (RFRT) to target the α_v_β_3_ integrin [83]. The nanoparticles can recognize the neoplastic endothelial cells specifically through binding affinity of multiple RGD ligands towards α_v_β_3_ integrin. After photoirradiation at a low dose, the permeabilization of vasculature in tumors is enhanced significantly and the drug release efficiency to tumor is increased by as much as 20.08-fold [83]. In addition, there are a lot of other hallmarks related to the tumor vasculature, which can be used in active targeting of PDT [67].

Tumor accumulation of PSs is an important step in PDT. Nevertheless, accelerated cellular internalization is the guarantee of effective PDT [97,98]. Tumor cell-targeted PDT is a responsible strategy to accelerate the phagocytosis/endocytosis of nanocarriers [51,99,100]. Nanocarriers grafted with targeting ligands can recognize and adhere to the surface receptors overexpressed by tumor cells and then improve the internalization ability. Numerous receptors can work as targets to develop internalization-prone drug delivery systems, such as folate (FA) receptor [101], epidermal growth factor receptor [90], transferrin receptor [102], and proteins/glycoproteins [103].

FA-targeted PDT can increase the cellular internalization via receptor-mediated endocytosis. Folic acid is an essential reactant in the synthesis process of nucleotide bases and folate receptors are overexpressed on the surfaces of the malignant cells. The stable, inexpensive, easily conjugated characteristics make folic acid a promise candidate ligands for targeted cancer therapy [85,104,105,106,107]. Cheng et al. developed a charge-switchable nanocapsules for FA-targeted chemo/photodynamic therapy [79]. The surface charge of nanocapsules can be switched to positive in mildly acidic conditions after the protonation of oleylamine and then facilitate the endocytosis by means of FA-targeting. Chang et al. reported a pH-responsive upconversion drug delivery systems for near infrared PDT [41]. The upconversion nanoparticle was grafted with FA, and then it was manufactured by loading PEGylated polymeric lipid vesicles (PLV). The nanocarrier maintains stable in blood circulation and enhances the accumulation in the tumor via the EPR effect. Once stimulated by mildly acidic conditions in tumor sites, the shell of PEGylated PLV is removed and the exposed FA ligand can increase cellular internalization.

Transferrin is a serum protein participating in the circulating iron transport through the blood into cells [7]. Transferring receptor is overexpressed on several types of cancer cells surfaces and it is closely correlated with cell growth, proliferation and metastasis [19]. Many drug delivery systems based on transferrin targeting have been developed for transporting PSs to target cells [102,108,109]. Yu et al. prepared a transferrin-modified nanoparticle loaded with hypocrellin A to enhance the antitumor efficacy of the PS [110]. The size distribution of the nanoparticle was around 96–156 nm in aqueous solution, which avoided the hydrophobic defect of PS and enhanced the ability of targeting to transferrin receptor. Animal experiments on A549 tumor-bearing model in nude mice showed that the drug delivery system can achieve remarkable tumor inhibition rate while slight side effects in normal organs. Actively targeting polymeric nanoparticles conjugated with a peptide (hTf) ligand were used for against triple-negative breast cancer (TNBC) [111]. The hTf can specifically bind to the transferrin receptor and promote internalization of the nanocarrier. This remarkable selective phototoxicity in TNBC cells implies that the transferrin-targeted nanocarrier is a promising platform for the treatment of TNBC.

**Figure 4:**
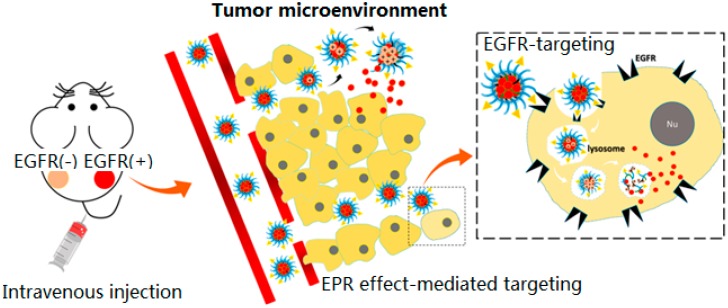
Schematic diagram of accumulation mechanisms of Ce6/GE11-(pH) micelle according to the EPR effect and EGFR targeting and successful Ce6 release in slightly acidic tumor microenvironments or lysosomes after EGFR-mediated endocytosis. Copyright (2018) American Chemical Society [116].

Epidermal growth factor (EGF) is a small polypeptide related to cell mitosis and angiogenesis [112]. Epidermal growth factor receptor (EGFR), a tyrosine kinase receptor, is overexpressed on many epithelial tumors cells surfaces [113,114,115]. This makes EGFR a potential important target for PDT [7,19,89]. Tan et al. designed an aptamer-based upconversion nanoparticles, which targeted the protein tyrosine kinase 7, for targeted PDT and bioimaging [82]. The sgc8 aptamer can specifically bind toward the EGFR and achieve highly efficient and selective cytotoxicity. Yang et al. developed a pH-responsive EGFR-targeting nanocarrier for PDT of colorectal cancer [116], and the mixed micelle contained pH-responsive copolymers and EGFR-targeting ligand. The nanocarrier was used to encapsulate hydrophobic Ce6 to enhance the photodynamic therapy effect. Biological experiments confirmed that mixed micelle can specifically target the colorectal cancer cells and significantly suppress tumor growth (Figure 4) [116].

CD44 receptor is a familiar target overexpressed on tumor cells, which can be specifically recognized by hyaluronic acid (HA) [117,118]. Nanoparticles based on hyaluronic acid was developed to encapsulate Ce6 and DOX for chemo-photodynamic therapy [119]. The nanocarriers can accumulate at the tumor site via EPR effect and be internalized rapidly for the active targeting of hyaluronic acid to the CD44. In endosomes or lysosomes, the inclusion was released for pH-responsive disassembly and enhanced the synergistic therapeutic efficacy accordingly [120].

There are many changes in physiology and biochemistry associated with the malignant cell transformation, such as steroids [121], bisphosphonates [122,123], α_v_ integrin receptors [4,53,54,124], and glycoproteins or lectins [103,125]. These changes can be designed as targets to enhance the specificity of cancer therapies [7].

Singlet oxygen species produced in type II processes are the most important ROS in PDT. However, the optimal effective radius of ^1^O_2_ can be negligible compared to the tumor cells (10^4^–10^5^ nm in diameter) [7], resulting in that elevated tumor cell internalization does not necessarily improve the effect of PDT. Therefore, subcellular targeting is another area worthy of exploration to improve the photodynamic response after cell internalization [48,126]. At present, subcellular targeting for mitochondria [127], endoplasmic reticulum [45,128], lysosome [126], and nucleus [46,129] has been developed with only a fairly limited number [48]. Among which, mitochondria targeting is of special concern.

**Figure 5:**
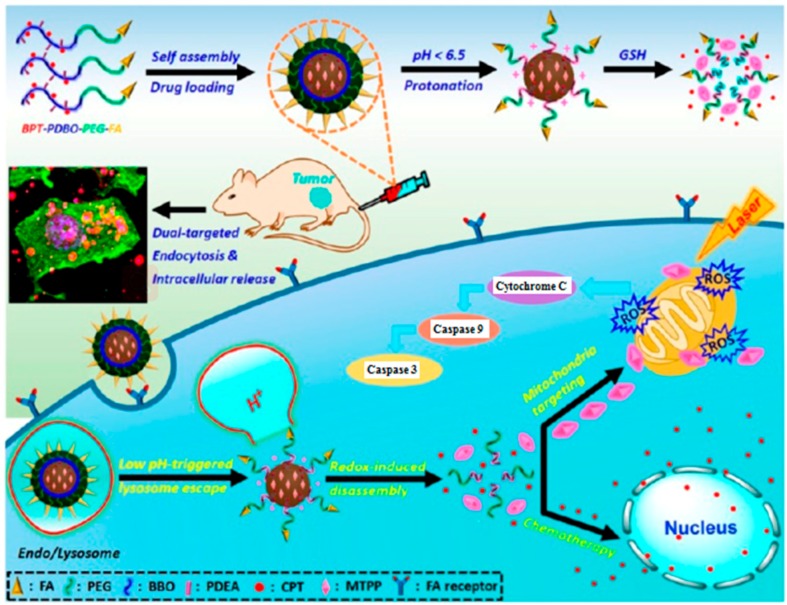
Illustration of cascade-responsive disassemble micelles with dual-targeting capability for tumor therapy in vivo. Copyright (2017) American Chemical Society [101].

Mitochondria, cellular organelle with a two-membrane structure, has high membrane potential (negative inside) and this makes it possible to target the mitochondria with cations, triphenylphosphonium (TPP) cation for example [63,130,131]. A smart drug delivery system with the function of tumor cell and mitochondria targeting was fabricated based on graphene oxide (NGO) [127]. Integrin α_v_β_3_ monoclonal antibody was used to modify NGO and target the tumor cells by the specific recognition between the integrin and its receptor on the cancer cells surfaces. Just as designed, the nanocarrier can effectively target the tumor cells and enhance cellular uptake. Once internalized, the modified NGO can escape from lysosomes and subsequently selectively accumulate in the mitochondria by electrostatic interaction with the negatively-charged mitochondria membrane. Cai et al. reported a pH and GSH cascade-responsive nanocarrier for dual-targeted chemo-photodynamic therapy [101]. Cationic porphyrin derivative, as the mitochondria-targeting PS, was encapsulated in polymeric micelle. The FA and camptothecin (CPT) were covalently conjugated with the polymeric micelle to modulate the biodistribution of drugs on systemic, local, and subcellular levels. After accumulating at tumor site via EPR effect, the prodrug nanocarrier can significantly enhance uptake efficiency by the folate receptor-mediated endocytosis. When escaping from lysosome, the CPT and PS are released in response to GSH in cytoplasm. Furthermore, the PS could selectively target mitochondria by electrostatic interaction and induce mitochondrial apoptotic, while the CPT could travel to the cell nucleus by diffusion and implement chemotherapeutic (Figure 5) [101].

Apart from the biodistribution, activation of PSs is another keypoint [132,133]. Although the PS can escape from the carrier and recover its activation after the biodegradation of polymer nanocarriers, stimuli responsive drug delivery systems are preferred [134,135,136,137,138,139].

### 2.2. Administer the Activation of PSs by Responses

Targeting strategies to restrict the localization of the PSs are not always effective for the unexpected payload leakage and retention at non-target sites during systemic circulation. Additionally, there is irreconcilable contradiction between enhancing targeting ability and reducing photosensitivity to skin and blood vessel: contradictory—needs for prolonging the retention time of the PSs in the blood against accelerating elimination from the blood circulation. Therefore, modulating the activation of PSs, which means the PSs can be activated and demonstrate phototoxicity only in the targeting site, is a reliable drug delivery strategy for PDT.

5-aminolevulinic acid (5-ALA) can be metabolized to protoporphyrin IX (PpIX) via heme biosynthetic enzymes in certain tumor cells [140,141,142]. The PpIX exhibits red fluorescence and can serve as a natural photosensitizer for PDT when activated with a light source of appropriate wavelength [143]. Now, the activatable 5-ALA, which can be orally administered, has been approved by European Agency for the Evaluation of Medicinal Products and FDA for the resection of malignant glioma in adults and treatment of actinic keratosis, respectively [16,31,140,142,143,144]. Image-guided photodynamic therapy based on 5-ALA offers an intriguing concept for the clinical trials of malignant gliomas recently [145]. Blood-brain barrier (BBB) can protect the central nervous system (CNS) by preventing passage of most harmful substances and circulating cells from entering the brain [146]. However, the BBB also makes the diseases of the brain and spinal cord the most dramatic disability in society for its shielding effect to the most of the beneficial drugs [147,148]. 5-ALA-based PDT, that can temporally increase the permeability or induce the disruption of the BBB, can enhance the anti-cancer effect by increasing permeability of the BBB to drugs [149,150].

Activatable PS formulations based on polymer nanocarriers should be able to switch between the deactivation and activation state [151]. In the deactivation state, the PSs can keep dormant in blood circulation and normal tissues, and demonstrate negligible phototoxicity even under illumination. After reactivated in response to the stimuli in tumor sites, the PSs recovery their activity and generate singlet oxygen to kill the cells directly when irradiated.

Many methods have been implemented to inhibit the generation of singlet oxygen, containing contact quenching, increasing the internal conversion, enhancing the Förster resonance energy transfer (FRET), accelerated dynamic quenching, and triplet state quenching [152], among which, quenching through the FRET is the most frequently used approach to maintain the deactivation state of the PS. FRET is an energy transfer process associated with space length (the distance between photosensitizer and its counterpart, quencher for example, should be in nanoscale) and spectral overlap (high overlap ratio between the absorptive spectral of chromophore acceptor and fluorescence emission spectral of PS is necessary) [153]. Stimuli-sensitive nanocarriers are ideal platforms to structure activatable PSs. They can control the activation of PSs by adjusting the distance of PS and quencher [154]. Many stimuli, such as external stimuli (e.g., light [155]) and internal environment of tumor (e.g., pH [135,139,156,157,158,159,160,161,162,163], enzyme [91,164], GSH [165,166,167,168], and ROS [169,170,171,172,173,174,175,176]), have been used as the keys to turn on the PSs by increasing the distance among the PSs.

Self-quenchable nanoparticles can control the off/on states of the PSs by regulating their aggregation/disaggregation [40,177]. The self-assembled nanoparticles tend to concentrate PSs into the nano-sized core and resulting in self-aggregation. When irradiated, the PS leaps into an excited singlet state from its ground state and then the neighboring PS molecules can quench the excited PS through energy transfer, resulting in the interruption of singlet oxygen generation [105,120,178,179]. After rapidly reversed from the suppression at the tumor site, the activated photosensitizers demonstrate effective singlet oxygen generation.

Chen et al. designed a plasma membrane activatable polymeric nanocarrier for enhanced photodynamic therapy [17]. The hydrophobic protoporphyrin IX (PpIX) and hydrophilic PEG were conjugated into biodegradable glycol chitosan (GC) and the polymer can self-assemble into nanocarriers in aqueous solution. The PSs in the inner core can be quenched effectively due to energy transfer. Once encountering plasma membranes, the PpIX moieties can insert into plasma membranes for their membrane affinity. The disassembling induced by plasma membranes can recover the activity of PpIX, leading to significantly enhanced phototoxicity. Glutathione (GSH) is much abundant in tumor intracellular environment (2–10 mM) and can be used to modulate the activation of PSs [180,181,182,183]. Huh et al. developed a GSH responsive bioactivatable delivery carrier for enhancing PDT [50]. The photosensitizers, pheophorbide a (PhA), were chemically conjugated to a biarmed methoxy poly(ethylene glycol) via disulfide bonds. The amphiphilic polymer prepared as aforesaid remains photoinactive in aqueous media due to the self-quenching effects from intramolecular and/or intermolecular. However, once getting inside the cells, the PhA were released instantaneously for the cleavage of the disulfide bonds induced by the GSH-rich intracellular environment in tumor cells. The activatable PSs can not only maximize the cancer treatment but also minimize the phototoxicity in normal tissues [166].

Similar to self-quenching, PS/quencher complexation is another alternative strategy for controlling and adjusting the photoactivity of PS sophisticatedly. Generally, photosensitizer should conjugate with a quencher through a bioactive linker.

**Figure 6:**
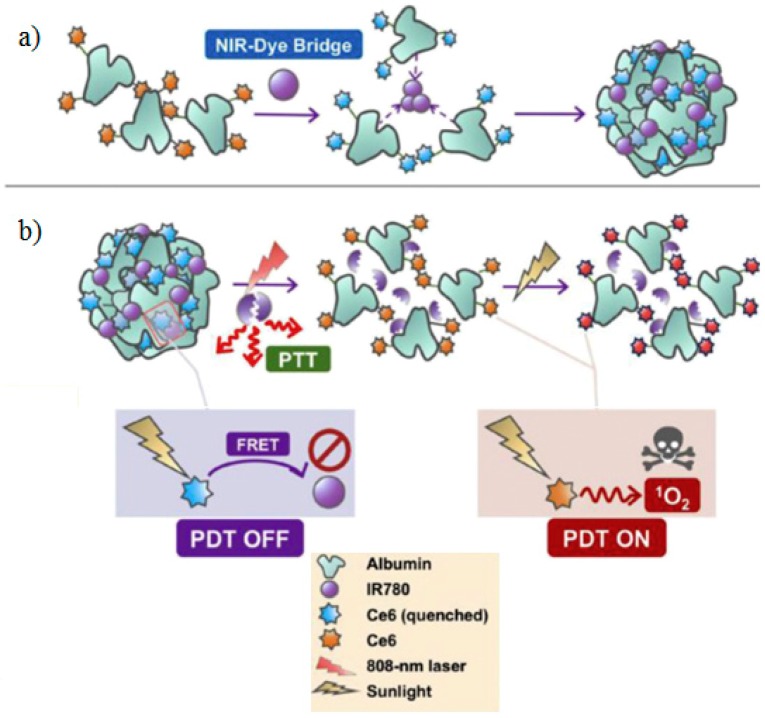
The scheme of switchable photodynamic therapy (Switch-PDT). (**a**) Schematic of self-assembly of albumin-Ce6 conjugates into NP induced by a NIR-dye bridge. (**b**) Schematic illustrating the quench effect of IR780 on Ce6 photosensitization (PDT OFF) and recovery of the photosensitization under 808-nm laser irradiation (PDT ON). Reprinted from Biomaterials, Yifan Zhang et al., Switchable PDT for reducing skin photosensitization by a NIR dye inducing self-assembled and photo-disassembled nanoparticles, 23–32, Copyright (2016), with permission from Elsevier [184].

NIR light is an excellent external stimulus to trigger the activity of PSs for its deep penetrability and exact controllability. Wu et al. designed a light-triggered switchable nanoparticle by quenching the Ce6 with near-infrared (NIR) dye IR-780 iodide (IR780) to reduce skin photosensitization in PDT [184]. The nanoparticle was fabricated by encapsulating the IR780 with self-assembling albumin-PS conjugates. Due to the good match of the spectrum, the pathway of Ce6 for singlet oxygen generation is suppressed by IR780 when in aggregation state, and the nanoparticle has no phototoxicity to the skin. After degradation of IR780 under NIR irradiation, the nanocarrier disaggregates and “turns on” the photosensitizer of the Ce6 (Figure 6).

pH-responsive polymers are preferred in the drug delivery systems. Jiang et al. conjugated Ce6 to gold nanorod (AuNR) via a pH responsive hydrazone bond for enhancing photothermal/photodynamic effect [185]. Before triggered, the Ce6 was quenched by the longitudinal surface plasmon resonance (LSPR) of the gold nanorod. After engulfed by the cancer cells, the hydrazone bond was cleaved upon the low pH in lysosomes and the Ce6 was separated from AuNR, recovering its phototoxicity and fluorescence. Meanwhile, as a potential photo-thermal therapy reagent, gold nanorod can translate the absorbed light of 808nm into heat and implement satisfactory photothermal therapy (PTT) effect [186,187].

**Figure 7:**
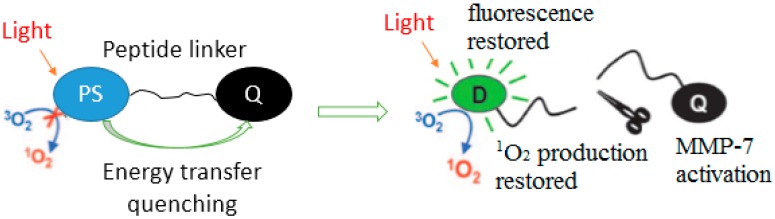
The concept of PMB. Copyright (2007) National Academy of Science [188].

Enzymes are conventional triggers in responsive drug delivery systems. Matrix metalloproteinase-7 (MMP7) is a hallmark of endothelial tumor cells and can cleave the certain peptide linker specifically [91,188]. Zheng et al. designed a photodynamic molecular beacons for activatable PDT by conjugating pyropheophorbide and black hole quencher 3 (BHQ3) with a MMP7-cleavable peptide linker. The activity of PS can be precisely controlled by MMP7, and the PS showed less phototoxicity until the peptide linker was cleaved in MMP7-expressing cells. This strategy can significantly minimize PDT complications and at the same time enhance the specificity and efficacy of PDT (Figure 7). Lange et al. have a series of excellent studies on drug activation by cancer associated enzymes for PDT [189,190,191]. It is one hot topic for 5-ALA and its esters to enhance their stability, reduce acute toxicity, and optimize systemic administration. Andrej Babič et al. designed a tunable phosphatase-sensitive stable 5-ALA derivatives by incorporating a phosphatase sensitive group to 5-ALA [192]. The prodrugs display controllable profiles of PpIX synthesis and fluorescence intensity.

## 3. Administration of Oxygen

In type II processes, oxygen is an indispensable element in PDT. The molecular oxygen can quench the excited triplet states of PS and subsequently generate highly cytotoxic singlet oxygen. However, the undesirable intrinsic tumor hypoxia in most tumor tissues, caused by uncontrollable tumor cell proliferation and imperfect vascular system, significantly affects the efficiency of PDT [193]. Even worse, oxygen consumption and tumor vasculatures shutdown during PDT will further aggravate tumor hypoxia, thus inducing angiogenesis, local invasive growth, and metastasis of cancers [58]. Currently, many efforts have been made to ameliorate or utilize the tumor hypoxia microenvironment to enhance the PDT efficiency.

### 3.1. Carrying Oxygen

Red blood cell (RBC), containing hundreds of millions of hemoglobin molecules, is a valid oxygen storage pool [194]. Zhang et al. reported a microcarriers based on RBC to overcome hypoxia and evade biological barriers in the process of PDT [43]. The UCNPs functionalized with hypoxia probe (HP) and PSs were installed into the surface of the RBC. The RBC conjugated with HP can implement site-specific O_2_ release in hypoxia cells only when the HP was activated by nitroreductases as well as excited with a 980 nm laser. With the increase of O_2_ supply, microcarriers exhibited enhanced PDT efficiency when exciting photosensitizer with 808 nm laser. This strategy controlling O_2_ release by NIR light and site-specific hypoxia probe is a promising means to improve the oxygen supply in PDT.

Perfluorocarbon is another candidate to carry oxygen for its highlighted oxygen capacity and extended ^1^O_2_ lifetime [195]. Due to the higher oxygen capacity, perfluorocarbon (PFC) nanodroplets can maintain a higher oxygen content even at hypoxic tumor microenvironment [10,196,197]. Photosensitizer loaded into PFC nanocarriers is isolated in oxygen self-enriched environment and could effectively enhance the producing of singlet oxygen. Beyond that, the longer ^1^O_2_ lifetime endows it with long-lasting photodynamic effects.

### 3.2. Oxygen Generations in Situ

Elevated level of endogenous hydrogen peroxide (H_2_O_2_) is a characteristic aberrance of cancer cells, and is associated with tumor aggressiveness and metastasis. H_2_O_2_ has been a possible source of oxygen through decomposing it into O_2_ to relieve tumor hypoxia [198]. Catalase, a specific catalytic enzyme, is considered as a promising candidate for triggering H_2_O_2_ decomposition [199]. Zhu et al. fabricated a chitosan based nanoparticles to load with catalase as an efficient catalyst via electrostatic interaction for enhanced photodynamic therapy [200]. The pH-sensitive aerobic nanoparticles can accumulate in tumors preferentially and exhibit rapid responsiveness to tumor acidic environment. The quick release of catalase could alleviate the hypoxia in solid tumors and enhance the PDT effect. Manganese dioxide (MnO_2_) is an ectogenic catalyzer which could reduce H_2_O_2_ and generate oxygen meanwhile [201]. When loaded with polymer, MnO_2_ could program oxygen generation rate and lower the acidity in the tumor microenvironment (from pH 6.7 to pH 7.2) [55,56]. Above all, the down-regulation of hypoxia-inducible factor-1 alpha and VEGF, induced by MnO_2_, can regulate the progression and aggressiveness of tumor cells [202].

### 3.3. Oxygen-Independent PDT

**Figure 8:**
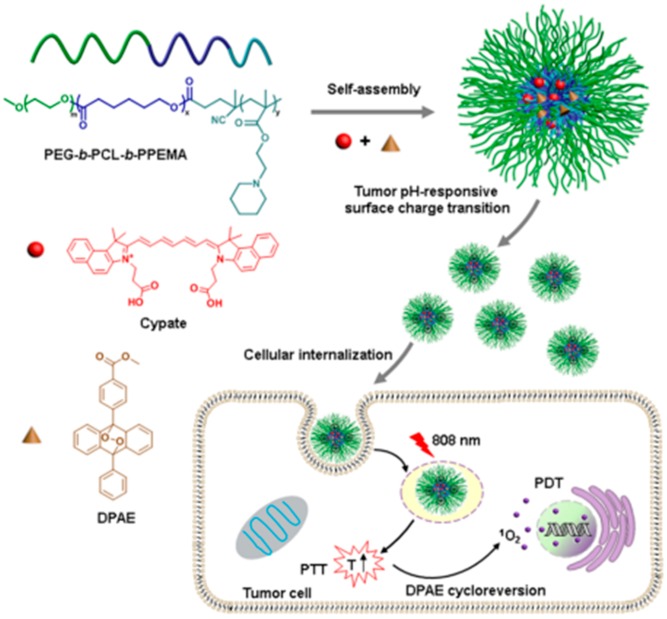
Schematic illustration of C/O@N-micelle with pH-responsive promoted cellular uptake and oxygen-independent photothermally triggered photothermal/photodynamic therapy under NIR irradiation. Reprinted from Journal of Controlled Release, 284, Yu Han et al., Oxygen-independent combined photothermal/photodynamic therapy delivered by tumor acidity-responsive polymeric micelles, 15–25, Copyright (2018), with permission from Elsevier [204].

Endoperoxide derivatives can generate ^1^O_2_ by chemical reaction, which is independent with the hypoxia microenvironment [203]. Ge et al. devised an oxygen-independent polymeric nanocarrier for combined PTT/PDT [204]. The nanoparticle was prepared by encapsulating the cypate and diphenylanthracene endoperoxide (DPAE, singlet oxygen donor) within a triblock copolymer. The nanocarrier had weak negative potential at pH 7.4 and showed remarkable tumor accumulation in tumor tissues due to prolong circulation time in the serum. After protonated in tumor tissues (at pH 6.8), the zeta-potentials turned to positive (+11 mV) and the internalization of the nanocarrier was accelerated. The cypate could induce remarkable hyperthermia under 808 nm NIR irradiation and implement potential PTT. Simultaneously, the DPAE went through thermal cycloreversion and generated singlet oxygen without participation of oxygen molecules in this process. This oxygen-independent combined PTT/PDT therapy strategy offers a rational opportunity to extend the category of PDT (Figure 8).

### 3.4. Utilizing Tumor Hypoxia

In distinction from improving the hypoxia microenvironment, one also can make much use of the tumor hypoxia physiology to design novel living delivery system. An azobenzene bridge is a hypoxia-responsive bond which can be severed in the tumor site [42]. After triggered in hypoxic environment, the PEGylation was shed and the cellular uptake of micelles was facilitated for the changes of the surface charge [44]. Tirapazamine (TPZ) is of selective toxicity to hypoxic tumor cells. PDT could activate TPZ by aggravating hypoxia through oxygen consumption and vascular shutdown effects [53].

## 4. Conclusions

PDT is a local therapy and represents an effective and highly selective therapeutic option in management of cancer. To optimize the PDT effect, comprehensive management the PSs, light, and tumor hypoxic microenvironment is the prerequisites. In addition, combination therapy with chemotherapy, photothermal therapy, surgery, and immunotherapies has been a particularly promising therapy mode for PDT.
